# Supplementary material for: The impact of preovulatory versus midluteal serum progesterone level on live birth rates during fresh embryo transfer
Source: PLoS One. 2021 Feb 11;16(2):e0246440. doi: 10.1371/journal.pone.0246440 (PMC7877612; doi:10.1371/journal.pone.0246440)
Supplement: S1 Table — N, 328 patients undergoing IVF/ICSI treatment. Rho, Spearman’s correlation coefficient. P < .05: Statistically significant. (DOC) [file pone.0246440.s001.doc]

**S1 Table.**

Spearman’s correlation between Preovulatory, Midluteal serum P_4_ concentration and Ovarian response

| **Variable** | **Estradiol_trigger** | **Follicles_trigger** | **Oocytes retrieved** | **Midluteal P_4_** |
| --- | --- | --- | --- | --- |
| *Preovulatory P_4_* Rho      *P* value | .42 | .33 | .30 | .15 |
|  | <.0001 | <.0001 | <.0001 | <.007 |

*N, 328 patients undergoing IVF/ICSI treatment. Rho, Spearman’s correlation coefficient.*

*P<.05: statistically significant*
