# Supplementary material for: The impact of preovulatory versus midluteal serum progesterone level on live birth rates during fresh embryo transfer
Source: PLoS One. 2021 Feb 11;16(2):e0246440. doi: 10.1371/journal.pone.0246440 (PMC7877612; doi:10.1371/journal.pone.0246440)
Supplement: S2 Table — *Chi-squared test for differences between mid-luteal serum P4 groups. P < .05: Statistically significant. NA, not applicable; OPU, Ovum pick-up; P4, progesterone (ng/mL). (DOC) [file pone.0246440.s002.doc]

**S2 Table.**

Relationship between Live birth rates and Midluteal (OPU+7) serum P_4_ quartiles

| **Variable** | **P_4_ Quartile 1**  **(<28)** | **P_4_ Quartile 2**  **(29-40)** | **P_4_ Quartile 3**  **(41-60)** | **P_4_ Quartile 4**  **(>60)** | **P-value*** | **Total** |
| --- | --- | --- | --- | --- | --- | --- |
| Number | 83 | 91 | 102 | 52 | NA | 328 |
| Live birth,  n (%) | 25 (30.12%) | 23 (25.27%) | 46 (45.10%) | 16 (30.77%) | .02 | 110 (33.5%) |

*Chi-squared test for differences between midluteal serum P_4_ groups. *P<.05: statistically significant*

*NA, not applicable; OPU, Ovum pick-up*; P_4_, progesterone (ng/mL)
