# Supplementary material for: The impact of preovulatory versus midluteal serum progesterone level on live birth rates during fresh embryo transfer
Source: PLoS One. 2021 Feb 11;16(2):e0246440. doi: 10.1371/journal.pone.0246440 (PMC7877612; doi:10.1371/journal.pone.0246440)
Supplement: S1 Dataset — (DOC) [file pone.0246440.s003.doc]

FILE #2

Dataset dictionary

The following file #2 (Word format) provides additional informations such as the variables names, the variables meanings, the measurement units and the missing data which might be helpful for the analysis of data included in the file #1 (Excel format)

- NA, not applicale (missing values)
- Age, female age (years);
- *BMI, body mass index;*
- *AFC, Antral Follicle Count;*
- *ting hormone; OPU, oocyte pick-up; P_4_ , progesterone(ng/ml); Q, quartile; standard deviation*
- *Infertility type (primary or secondary)*
- *Infertility duration (years)*
- *FSH-D2, follicle-stimulating hormone on day 2 of cycle*
- *LH-D2, luteinizing hormone on day 2 of cycle*
- *E2_D2, estradiol (pg/ml) on day 2 of cycle*
- Antagonist total dose(mg)
- Duration of stimulation (days)
- E2_trigger, estradiol (pg/ml) on the day of GnRH agonist trigger
- P4_ trigger, progesterone (ng/ml) on the day of GnRH agonist trigger
- LH_trigger, *luteinizing hormone (IU/ml)*  on the day of GnRH agonist trigger
- Follicle#, number of follicles on the day of GnRH agonist trigger
- Endometrium, thickness of endometrium (mm) on the day of GnRH agonist trigger
- COC #, number of oocytes with oophorus cumulus
- Oocytes M2 #, number of mature oocytes
- Zygote #, number of fertilized oocyte
- Embryo #, number of total embryos
- Cryopreserved embryos #, number of embryos frozen
- Embryos transferred #, number of embryos transferred
- Day of embryos transferred, day 2 or 3 after oocyte pick-up
- TMS, sperm migration test
- Sperm motility (a+b), percentage of sperm that exhibit rapid and progressive movement
- GnRH_trigger (1, 0), patient who received (1) or not (0) an additional dose of GnRH agonist (Triptorlin 0.1 mg) on day 6 after Oocyte pick up
- FSH-OPU+7, FSH (IU/L) on day 7 after oocyte pick-up
- LH OPU+7, LH FSH (IU/L) on day 7 after oocyte pick-up
- E2_OPU +7, estradiol on day 7 after oocyte pick-up
- P4_OPU+7, progesterone on day 7 after oocyte pick-up (midluteal P4)
- ß-HCG (1, 0), positive or negative serum pregnancy test on day 14 after oocyte pick up
- ß-HCG level, ß-HCG serum concentration (mIU/ml) on day 14 after oocyte pick up
- Gest_sacs #, number of gestational sacs detected by ultrasound 5 weeks gestation
- Early Pregnancy loss, pregnancy loss before 5 weeks of gestation
- ongoing pregnancy, pregnancy with positive heart activity beyond 12 weeks of gestation
- Live birth, a delivery of a live baby beyond 26 weeks after oocyte pick up
- P_4_ -trigger-Quartiles, serum P_4_ levels on the day of trigger converted from continuous variables into categorical variables by dividing them into four groups (quartiles) based on 25^th^, 50^th^ and 75^th^ percentiles. Q1 included 0–25%, Q2 included 25–50%, Q3 included 50–75% and Q4

[Q1: <0.74, Q2: 0.75–0.98, Q3: 0.99-1.30, and Q4: > 1.30 ng/mL]. Conversion factor

to SI unit, 3.180.

- Midluteal-P4-Quartiles, serum P_4_ levels on the day 7 after oocyte pick up converted from continuous variables into categorical variables by dividing them into four groups (quartiles) based on 25^th^, 50^th^ and 75^th^ percentiles. Q1 included 0–25%, Q2 included 25–50%, Q3 included 50–75% and Q4
- [Q1: <28, Q2: 29–40, Q3: 41-60, and Q4: > 60 ng/mL]. Conversion factor to SI unit, 3.180.
- Follicles-Categories, follicles> 11 mm on the day of GnRH agonist trigger were divided into three categories according to the ovarian response ; low response (< 6 follicles), intermediate response (6-18) and high ovarian response (>18).
- LH Quartiles-trigger, serum LH (IU/L) levels on the day of GnRH agonist trigger were divided into four categories according to the quartiles (Q1-Q4). (Q1: <0.68, Q2: 0.69-0.98, Q3: 0.99-1.6, Q4: > 1.6 )

Abdelhamid Benmachiche (AB)

Corresponding Author

benmachiche@gmail.com
