# Supplementary material for: The impact of preovulatory versus midluteal serum progesterone level on live birth rates during fresh embryo transfer
Source: PLoS One. 2021 Feb 11;16(2):e0246440. doi: 10.1371/journal.pone.0246440 (PMC7877612; doi:10.1371/journal.pone.0246440)
Supplement: Attachment — Submitted filename: Response to reviewers.doc [file pone.0246440.s006.doc]

**Dear Editor**

We appreciate the effort and the time that you and the reviewers have dedicated to providing your valuable feedback on our manuscript.

We believe that we had addressed all the questions and concerns raised by the editor as well as the reviewer #2.

We have included the page and line numbers in the revised manuscript to help the reviewer identify our changes.

We look forward to hearing from you regarding our revision submission and to respond to any further questions and comments you may have.

Abdelhamid Benmachiche (AB)

Corresponding Author

**RESPONSE TO COMMENTS FROM THE EDITOR AND REVIEWER #2**

I. **Editor**

**Data availability**

According to **The PLOS Data Policy**, the authors are required to make all data underlying the findings described in their manuscript fully available without restriction. Three main options are being recommended; the data should be provided as part of the manuscript **or** its supporting information, **or** deposited to a public repository.

With your permission and for clarification, we take this opportunity to emphasize that the authors confirm that the relevant data supporting the findings of this manuscript are fully available in its **supporting information files** **without any restriction**;

S3 Dataset (XLSX)_File 1 provides data within a spreadsheet (Excel format).

S4 Dataset (DOCX)_File 2 (Word format) gives additional details such as the variables names, the variables meanings, the measurement units and the missing data which might be helpful for the analysis of data included in S3 Dataset (XLSX)_File 1.

🡪 Please see ***Supporting information files,*** page 16 lines 349 and 350 of the revised manuscript.

Thus, we believe that we have totally complied with the **PLOS Data Policy**

II. **Reviewer #2**

**Question #1**

*Please remove some remaining sentences on prediction analysis—lines 124-125 in Statisticis section; the second sentence in the Discussion section.*

**Response #1**

We completely agree with this and have, accordingly, incorporated the requested changes, that is, all sentences on prediction analysis have been removed from the revised manuscript.

.

🡪 Please see *Statistic section*, page 6 line 126 and *Discussion section*, page 12 lines 249-251 of the revised manuscript.

We deeply appreciate the reviewer’s input and insightful comments
